# Differences in Colistin Administration and Bacterial and Treatment Outcomes in Critically Ill Patients

**DOI:** 10.1038/s41598-019-44965-y

**Published:** 2019-06-19

**Authors:** Sunmi Jung, Eun Kyoung Chung, Min Sun Jun, Eun Sun Son, Sandy Jeong Rhie

**Affiliations:** 1 Graduate School of Clinical Health Sciences, Ewha Womans University, Seoul, 03760 Republic of Korea; 2 Division of Pharmacy, Yonsei University Health System, Seoul, 03722 Republic of Korea; 3 College of Pharmacy, Kyung Hee University, Seoul, 02447 Republic of Korea; 4 Department of Pharmacy, Kyung Hee University Hospital at Gangdong, Seoul, 05278 Republic of Korea; 5 College of Pharmacy, Ewha Womans University, Seoul, 03760 Republic of Korea; 6 Division of Life & Pharmaceutical Sciences, Ewha Womans University, Seoul, 03760 Republic of Korea; 7 Department of Pharmacy, Ewha Womans University Mokdong Hospital, Seoul, 07985 Republic of Korea

**Keywords:** Bacterial infection, Outcomes research, Risk factors

## Abstract

The desired target steady-state average colistin concentration (C_ss,avg_) to balance between therapeutic effectiveness and nephrotoxicity is largely unclear. The objective of this study was to evaluate the effect of the desired target colistin C_ss,avg_ on the effectiveness and safety of IV colistin therapy in critically ill patients. Overall, 153 critically ill patients (71% males) receiving IV colistin were retrospectively analyzed. The desired target colistin C_ss,avg_ was estimated based on the daily colistin dose and creatinine clearance of each patient. No significant predictor for clinical cure was identified. However, microbiological outcome was significantly associated with pneumonia compared to bacteremia (odds ratio [OR] 0.092, 95% confidence interval [CI] [0.033–0.251], *P* < 0.001) and the use of IV colistin loading dose (OR 2.783, 95% CI [1.126–6.880], *P* = 0.027). Colistin-associated nephrotoxicity was significantly less likely to occur in patients who received inhaled colistin close to the time of IV colistin therapy (OR 0.331, CI [0.119–0.925], *P* = 0.035). The desired target C_ss,avg_ of colistin was not associated with treatment outcomes or the risk of nephrotoxicity. Loading dose and inhaled colistin use near the time of IV colistin therapy may be considered to maximize therapeutic effectiveness and minimize the risk of colistin-associated nephrotoxicity, respectively.

## Introduction

Treatment of infections caused by multidrug-resistant (MDR) gram-negative bacterial pathogens is challenging due to limited treatment options^1^. Due to altered physiological characteristics related to critical illness and the care provided in an intensive care unit (ICU) such as common use of broad-spectrum antibiotic agents and invasive procedures, critically ill patients are more susceptible to MDR bacterial infections associated with substantially increased morbidity and mortality^2–6^. Globally, the most clinically significant MDR bacterial pathogens include *Acinetobacter baumannii* and *Pseudomonas aeruginosa*^2,7^. Some strains of these MDR pathogens are resistant to nearly all antimicrobial agents including aminoglycosides, cephalosporins, fluoroquinolones, and carbapenems, leaving very few antibiotic options for the treatment of infections caused by these organisms^2^. Among the limited therapeutic options, colistin is one of the most commonly used antibacterial medication for the treatment of life-threatening invasive infections caused by MDR pathogens^2,8^.

Colistin is a polymyxin antimicrobial agent, specifically polymyxin E^9,10^. It is intermittently infused via an intravenous (IV) route as a prodrug called colistin methanesulfonate (CMS)^9,10^. It is rapidly bactericidal with a substantial postantibiotic effect against gram-negative organisms including *Acinetobacter baumannii*, *Pseudomonas aeruginosa*, and *Klebsiella* species^9,11^. The bactericidal activity of colistin appears concentration-dependent; the ratio of the area under the unbound plasma concentration-time curve over a dosing interval to minimum inhibitory concentration (*f*AUC:MIC) was suggested to be the pharmacokinetic-pharmacodynamic parameter most predictive of colistin activity^9,12,13^. According to a previous study using the animal model infected by *Pseudomonas aeruginosa*, a colistin *f*AUC:MIC of 12 to 48 was associated with near-optimal to optimal bacterial killing^12^. However, accurate estimation of *f*AUC requires serial collection of blood samples over a dosing interval, which is not feasible in routine clinical practice. Therefore, the *f*AUC is commonly expressed as the average steady-state plasma concentration, which is *f*AUC divided by 12 hours (typical one dosing interval for patients with normal renal function). To achieve the colistin target of *f*AUC:MIC of 12 to 48 for an organism with an MIC of 1 mg/L, the *f*AUC:MIC of 12 to 48 correspond to the target average steady-state total plasma colistin concentrations (C_ss,avg_) of 1 to 4 mg/L. This target C_ss,avg_ was not considered in the current dosages approved by the Food and Drug Administration (FDA) in the United States: 2.5 to 5 mg/kg daily in 2 to 4 divided doses for patients with normal renal function with dosage adjusted based on the renal function of each patient^14^. More recently, the target C_ss,avg_-driven colistin dosing algorithms were developed by modeling and simulation approaches^15,16^. Currently, many clinicians use these dosing algorithms to determine colistin dose for each individual patient for optimal systemic colistin exposure. However, the target colistin C_ss,avg_ range is relatively wide for dosing calculation, and it has not been prospectively validated in large-scale human studies. Moreover, although controversial, the potential association between the risk of nephrotoxicity and the colistin C_ss,avg_ of ≥2.5 mg/L further complicates the clinical decision to choose the appropriate colistin dosing^17–19^. Consequently, the target colistin C_ss,avg_ value for dosing calculation to optimally balance between clinical effectiveness and nephrotoxicity is largely unclear, resulting in highly variable colistin dosages used in clinical practice^13,15,16,19,20^.

Although colistin pharmacokinetics have been extensively studied in various patient populations, to our knowledge, the relationship between the desired target colistin C_ss,avg_ for colistin dosing and the treatment outcomes in critically ill patients with acute infections has not been elaborated. Therefore, the objective of this study was to evaluate the effect of the desired target colistin C_ss,avg_ for dosing on the effectiveness and safety of IV colistin therapy in critically ill patients.

## Results

**Figure 1:**
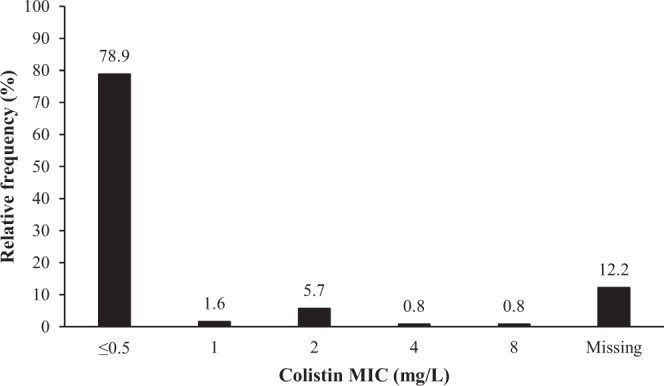
Distribution of the minimum inhibitory concentrations (MICs, mg/L) for colistin (n = 108).

A total of 170 patient records were identified to meet the inclusion and exclusion criteria. Due to the limited number of patients with infectious diseases other than pneumonia and bacteremia (n = 4 with urinary tract infection, n = 9 with intra-abdominal infection, n = 2 with skin and soft tissue infection), only the patients with pneumonia (n = 114) and bacteremia (n = 41) were included in the analysis cohort. In terms of causative pathogens, *Klebsiella pneumoniae* caused pneumonia in two patients only, and thus, these patients were excluded from our final analysis cohort. Overall, our final analysis cohort included 153 patients (Table 1). Loading dose of IV colistin was administered to 80 patients (52%). The median (range) desired target colistin C_ss,avg_, which was estimated using Eqs 1 and 2 constructed in a previous study, was 3.10 (2.24–7.24) mg/L from loading doses and 3.17 (0.37–15.56) mg/L from maintenance doses:

1 $${{\rm{C}}}_{{\rm{ss}},{\rm{avg}}}=\frac{{\rm{Loading}}\,{\rm{dose}}\,{\rm{of}}\,{\rm{colistin}}\,{\rm{base}}\,\text{activity}\,({\rm{mg}})}{2\times {\rm{body}}\,{\rm{weight}}({\rm{kg}})}$$

2 $${{\rm{C}}}_{{\rm{ss}},{\rm{avg}}}=\frac{{\rm{Daily}}\,{\rm{dose}}\,{\rm{of}}\,{\rm{colistin}}\,{\rm{base}}\,{\rm{activity}}\,({\rm{mg}})}{[(1.50\times {{\rm{CrCl}}}_{{\rm{n}}})+30]}$$

where body weight is the lower of ideal body weight (IBW) or total body weight (TBW) in kg, and CrCl_n_ is the estimated creatinine clearance (CrCl) normalized to calculated body surface area at baseline in mL/min/1.73 m^2^ ^15,21,22^. Treatment outcome data were missing in 30 patients each for clinical response and microbiological eradication. In the patients with treatment outcome data, most of the causative organisms had a colistin MIC of ≤0.5 mg/L (n = 97; 97/123 = 79%), which was determined by the Vitek 2 AST N212 card for nonfermenters in the Vitek 2 automated system (bioMerieux, Durham, NC, USA) (Fig. 1). Table 2 summarizes antimicrobial susceptibilities of the cultured isolates of *Acinetobacter baumannii* and *Pseudomonas aeruginosa*. All of the clinical cultured isolates were susceptible to colistin with the exception of three *Acinetobacter baumannii* isolates; they were resistant to colistin. Bacterial susceptibility data were not available in 15 patients. Overall, a total of 123, 108, and 153 patient records were included in the analysis of clinical cure, microbiological eradication, and colistin-associated nephrotoxicity, respectively.

**Table 1:** Patient characteristics (n = 153).

Characteristics	Mean ± standard deviation or median (range) unless otherwise stated
Age (years)	66 (21–91)
Male sex (No.)	109 (71%)
Height (cm)	165 (125–186)
Weight (kg)	57 (37–99)
Body mass index (kg/m^2^)	21.5 ± 4.1
Creatinine clearance at the beginning of therapy (mL/min)	55 ± 19
Disease severity score	
Charlson comorbidity index	2 (0–12)
APACHE II^a^	24 (2–42)
Infectious diseases	
Pneumonia (No.)	112 (73%)
Bacteremia (No.)	41 (27%)
Causative organism	
*Acinetobacter baumannii* (No.)	121 (79%)
*Pseudomonas aeruginosa* (No.)	32 (21%)
No. of concurrently used antibiotics other than intravenous colistin	3 (0–9)^b^
Use of inhaled colistin therapy immediately prior to the initiation or after the end of intravenous colistin treatment	19 (12%)
Concomitant antibacterials (No.)	
Piperacillin-Tazobactam	123 (80%)
Third generation cephalosporins	130 (85%)
Fourth generation cephalosporins	128 (84%)
Aminoglycosides	16 (10%)
Glycopeptides	139 (91%)
Rifampin	26 (17%)
Fluoroquinolones	46 (30%)
Sulfonamides	55 (36%)
No. of concurrently used nephrotoxins other than intravenous colistin	3 (1–5)
Concurrent nephrotoxins other than antibacterials	
Vasopressors	139 (91%)
Diuretics	141 (92%)
Intravenous contrast media	23 (15%)
Polyene	57 (37%)
Loading dose of intravenous colistin^c^ (mg)	300 (225–990)
Maintenance dose of intravenous colistin (mg)	300 (75–1,080)
Average daily dose of intravenous colistin (mg)	312 (84–1,004)
Per ideal body weight (mg/kg)	5.4 (1.5–16.0)
Per total body weight (mg/kg)	5.7 (1.2–21.0)
Cumulative intravenous colistin dose (mg)	4,500 (700–94,220)
Duration of intravenous colistin therapy (days)	14 (3–156)

^a^Acute Physiology and Chronic Health Evaluation II (APACHE II) score ranging from 0 to 71 with higher scores corresponding to more severe disease and increased risk of death; data available in 82 patients only.

^b^Only 2 patients were treated with intravenous colistin monotherapy.

^c^Only 80 patients (52%) received loading dose.

**Table 2:** Antimicrobial susceptibilities of the cultured clinical isolates (n = 108^a^).

*Acinetobacter baumannii* (n = 85)		*Pseudomonas aeruginosa* (n = 23)	
Antimicrobial agents	No. of susceptible isolates (%)	Antimicrobial agents	No. of susceptible isolates (%)
Ampicillin	18 (21)	Amikacin	16 (70)
Ampicillin/sulbactam	7 (8)	Aztreonam	3 (13)
Ceftazidime	0 (0)	Ceftazidime	3 (13)
Ciprofloxacin	0 (0)	Ciprofloxacin	2 (9)
Colistin	82 (96)	Colistin	23 (100)
Cefepime	1 (1)	Cefepime	4 (17)
Cefotaxime	0 (0)	Gentamicin	8 (35)
Gentamicin	8 (9)	Imipenem	2 (9)
Imipenem	1 (1)	Levofloxacin	1 (4)
Levofloxacin	0 (0)	Meropenem	2 (9)
Meropenem	1 (1)	Piperacillin	2 (9)
Minocycline	66 (78)	Piperacillin/tazobactam	3 (13)
Piperacillin	0 (0)		
Piperacillin/tazobactam	0 (0)		
Sulfamethoxazole/trimethoprim	11 (13)		
Tigecycline	71 (84)		

^a^Data to evaluate microbiological response and bacterial susceptibilities were missing in 30 and 18 patients, respectively, and data for both were missing in three patients; therefore, antimicrobial susceptibility data were collected from 108 patient records only.

In the univariate analysis, clinical cure was more likely to occur in patients with bacteremia, those receiving loading dose, those receiving lower cumulative dose, and those treated for a shorter period of time (Table 3). For the microbiological outcome, microbiological eradication was more likely to be achieved in patients with higher baseline CrCl, those with bacteremia, those infected with *Acinetobacter baumannii*, those receiving an aminoglycoside concurrently, those who received IV colistin loading dose, and those treated for a shorter period of time (Table 4). In terms of safety, colistin-induced nephrotoxicity was more likely to occur in patients with lower baseline CrCl, those not receiving inhaled colistin therapy immediately prior to the initiation or after the end of systemic colistin treatment, those receiving smaller cumulative IV colistin dose, and those treated for a shorter period of time (Table 5). Dose-related factors of colistin such as colistin maintenance dose and the desired target C_ss,avg_ were not significantly associated with colistin treatment outcomes. Based on the multiple logistic regression analysis, none of the tested factors were significantly associated with clinical cure (Table 3). However, microbiological eradication was significantly associated with the infectious indication (odds ratio [OR] with 95% confidence interval [CI]: 0.092 [0.033–0.251] for pneumonia compared to bacteremia, *P* < 0.001) and the use of IV colistin loading dose (OR with 95% CI: 2.783 [1.126–6.880], *P* = 0.027) (Table 4). Colistin-induced nephrotoxicity, assessed using the Acute Kidney Injury Network (AKIN) criteria, was significantly less likely to occur in patients who received inhaled colistin treatment immediately prior to the initiation or after the end of systemic colistin therapy (OR with 95% CI: 0.331 [0.119–0.925], *P* = 0.035) (Table 5)^23^.

**Table 3:** Factors evaluated for the association with clinical cure (n = 123^a^).

Characteristics	Univariate analysis^b,c^			Multivariable analysis	
	Clinical cure (n = 43)	Clinical failure (n = 80)	*P* value	Odds ratio (95% CI)	*P*-value
Age (years)	64 (23–90)	66 (21–91)	0.208	N/E	N/E
Male sex (No.)	27 (63%)	57 (71%)	0.336	N/E	N/E
Weight (kg)	55.2 (40.6–80.5)	56.9 (37.5–99.2)	0.561	N/E	N/E
Body mass index (kg/m^2^)	21.0 ± 4.1	21.7 ± 3.9	0.352	N/E	N/E
Creatinine clearance (CrCl) at the beginning of therapy^d^ (mL/min)	54 ± 17	57 ± 19	0.352	N/E	N/E
Charlson comorbidity index	2 (0–10)	2.5 (0–12)	0.529	N/E	N/E
Infectious diseases			**0.082**		0.099
Pneumonia (No.)	**27 (63%)**	**62 (78%)**			
Bacteremia (No.)	**16 (37%)**	**18 (22%)**			
Causative organism			0.614		N/E
*Acinetobacter baumannii* (No.)	36 (84%)	64 (80%)		N/E	
*Pseudomonas aeruginosa* (No.)	7 (16%)	16 (20%)		N/E	
No. of concurrent antibiotics used other than intravenous colistin	3 (1–9)	4 (1–9)	0.803	N/E	N/E
Use of inhaled colistin immediately prior to the initiation or after the end of systemic colistin treatment (No.)	7 (16%)	11 (14%)	0.705	N/E	N/E
Concurrent antimicrobials (No.)					
Piperacillin-tazobactam	11 (26%)	16 (20%)	0.476	N/E	N/E
Third generation cephalosporins	7 (16%)	15 (19%)	0.733	N/E	N/E
Fourth generation cephalosporins	7 (16%)	17 (21%)	0.507	N/E	N/E
Aminoglycosides	6 (14%)	8 (10%)	0.510	N/E	N/E
Rifampin	5 (12%)	19 (24%)	0.106	N/E	N/E
Fluoroquinolones	13 (30%)	30 (38%)	0.420	N/E	N/E
Sulfonamides	19 (44%)	27 (34%)	0.254	N/E	N/E
Use of intravenous colistin loading dose (No.)	**26 (60%)**	**34 (42%)**	**0.057**		0.068
Maintenance dose of intravenous colistin (mg)	337.5 (75–1080)	300 (100–900)	0.426	N/E	N/E
Desired target colistin C_ss,avg_ estimated from the maintenance dose^e^ (mg/L)	2.95 (0.37–15.56)	3.175 (0.78–6.29)	0.701	N/E	N/E
Desired target colistin C_ss,avg_^e^ ≥ 2 mg/L (No.)	33 (77%)	62 (78%)	0.924	N/E	N/E
Average daily dose of intravenous colistin (mg)	321 (84–1004)	300 (92–588)	0.414	N/E	N/E
Per ideal body weight (mg/kg)	5.4 (1.5–16.0)	4.9 (1.5–13.8)	0.570	N/E	N/E
Per total body weight (mg/kg)	6.2 (2.1–21.0)	5.4 (1.2–13.7)	0.319	N/E	N/E
Cumulative intravenous colistin dose (mg)	**4225 (1350–94220)**	**5802 (925–43800)**	**0.085**	N/E	N/E
Duration of intravenous colistin therapy (days)	**14 (4–156)**	**17.5 (4–146)**	**0.047**	N/E	N/E

Abbreviation: C_ss,avg_, average steady-state plasma concentration; CI, confidence interval; N/E, not estimated.

^a^Data to determine clinical cure were not available in 30 patients, so 123 patient records were included in the analysis.

^b^Mean ± standard deviation or median (range) unless otherwise noted.

^c^Bolded indicate factors significantly associated with clinical cure.

^d^Estimated by the Cockcroft-Gault equation using the actual measured serum creatinine concentration (SCr) if SCr was ≥1 mg/dL and the lower of ideal body weight (IBW) or total body weight (TBW); if the patient’s SCr was <1 mg/dL, SCr was rounded up to 1 mg/dL for CrCl estimation.

^e^Estimated by ${{\rm{C}}}_{{\rm{ss}},{\rm{avg}}}=\frac{{\rm{Daily}}\,{\rm{dose}}\,{\rm{of}}\,{\rm{colistin}}\,{\rm{base}}\,{\rm{activity}}\,({\rm{mg}})}{[(1.50\times {{\rm{CrCl}}}_{{\rm{n}}})+30]}$ where CrCl_n_ is CrCl normalized to body surface area estimated by the Mosteller method at baseline in mL/min/1.73 m^2^.

**Table 4:** Factors evaluated for the association with microbiological eradication (n = 108^a^).

Characteristics	Univariate analysis^b,c^			Multivariable analysis^c^	
	Microbiological eradication (n = 48)	Microbiological failure (n = 60)	*P* value	Odds ratio (95% CI)	*P* value
Age (years)	64 (31–83)	66 (21–90)	0.581	N/E	N/E
Male sex (No.)	34 (71%)	41 (68%)	0.779	N/E	N/E
Weight (kg)	55.6 (42.0–99.2)	56.6 (37.5–76.5)	0.358	N/E	N/E
Body mass index (kg/m^2^)	21.3 ± 4.1	21.5 ± 3.9	0.736	N/E	N/E
Creatinine clearance (CrCl) at the beginning of therapy^d^ (mL/min)	**60 ± 19**	**55 ± 16**	**0.094**		0.373
Charlson comorbidity index	2 (0–12)	2.5 (0–11)	0.146	N/E	N/E
Susceptibility			0.406		N/E
MDR strains susceptible to colistin only (No.)	8 (17%)	5 (8%)		N/E	
MDR strains susceptible to ≥2 antibacterial agents including colistin (No.)	39 (81%)	54 (90%)		N/E	
MDR strains resistant to colistin (No.)	1 (2%)	1 (2%)		N/E	
Infectious diseases			**<0.001**		**<0.001**
Pneumonia (No.)	**20 (42%)**	**53 (88%)**		**0.092 (0.033–0.251)**	
Bacteremia (No.)	**28 (58%)**	**7 (12%)**		**1 (reference)**	
Causative organism			**0.014**		0.360
*Acinetobacter baumannii* (No.)	**43 (90%)**	**42 (70%)**			
*Pseudomonas aeruginosa* (No.)	**5 (10%)**	**18 (30%)**			
No. of concurrent antibiotics used other than intravenous colistin	4 (1–9)	4 (1–8)	0.451	N/E	N/E
Use of inhaled colistin immediately prior to the initiation or after the end of systemic colistin treatment (No.)	7 (15%)	10 (17%)	0.768	N/E	N/E
Concurrent antimicrobials (No.)					
Piperacillin-tazobactam	15 (31%)	12 (20%)	0.180	N/E	N/E
Third generation cephalosporins	9 (19%)	11 (18%)	0.956	N/E	N/E
Fourth generation cephalosporins	10 (21%)	12 (20%)	0.915	N/E	N/E
Aminoglycosides	**9 (19%)**	**4 (7%)**	**0.055**		0.115
Rifampin	11 (23%)	9 (15%)	0.293	N/E	N/E
Fluoroquinolones	20 (42%)	19 (32%)	0.282	N/E	N/E
Sulfonamides	21 (44%)	20 (33%)	0.268	N/E	N/E
Use of intravenous colistin loading dose (No.)	**29 (60%)**	**22 (37%)**	**0.014**	**2.783 (1.126–6.880)**	**0.027**
Maintenance dose of intravenous colistin (mg)	360 (120–900)	75 (300–1080)	0.432	N/E	N/E
Desired target colistin C_ss,avg_ estimated from the maintenance dose^e^ (mg/L)	3.18 (1.03–8.29)	2.90 (0.37–15.56)	0.826	N/E	N/E
Desired target colistin C_ss,avg_^e^ ≥ 2 mg/L (No.)	38 (79%)	46 (77%)	0.756	N/E	N/E
Average daily dose of intravenous colistin (mg)	341 (92–733)	300 (97–1004)	0.824	N/E	N/E
Per ideal body weight (mg/kg)	5.4 (1.5–11.6)	5.2 (1.7–16.0)	0.931	N/E	N/E
Per total body weight (mg/kg)	6.1 (1.2–13.7)	5.7 (2.0–21.0)	0.574	N/E	N/E
Cumulative intravenous colistin dose (mg)	4655 (1040–33684)	5888 (925–94220)	0.119	N/E	N/E
Duration of intravenous colistin therapy (days)	**14 (4–134)**	**18 (4–156)**	**0.023**		0.313

Abbreviations: C_ss,avg_, average steady-state plasma concentration; CI, confidence interval; MDR, multi-drug resistant; N/E, not estimated.

^a^Data to evaluate microbiological response and bacterial susceptibility were missing in 30 and 18 patients, respectively, and data for both were missing in three patients; therefore, only 108 patient records were included in the analysis of microbiological eradication.

^b^Mean ± standard deviation or median (range) unless otherwise noted.

^c^Bolded indicate factors significantly associated with microbiological eradication.

^d^Estimated by the Cockcroft-Gault equation using the actual measured serum creatinine concentration (SCr) if SCr was ≥1 mg/dL and the lower of ideal body weight (IBW) or total body weight (TBW); if the patient’s SCr was <1 mg/dL, SCr was rounded up to 1 mg/dL for CrCl estimation.

^e^Estimated by ${{\rm{C}}}_{{\rm{ss}},{\rm{avg}}}=\frac{{\rm{Daily}}\,{\rm{dose}}\,{\rm{of}}\,{\rm{colistin}}\,{\rm{base}}\,\mathrm{activity}\,({\rm{mg}})}{[(1.50\times {{\rm{CrCl}}}_{{\rm{n}}})+30]}$ where CrCl_n_ is CrCl normalized to body surface area estimated by the Mosteller method at baseline in mL/min/1.73 m^2^.

**Table 5:** Factors evaluated for the association with nephrotoxicity (n = 153).

Characteristics	Univariate analysis^a,b^			Multivariable analysis^b^	
	Nephrotoxicity (n = 84)	No nephrotoxicity (n = 69)	*P* value	Odds ratio (95% CI)	*P* value
Age (years)	66 (23–90)	66 (21–91)	0.504	N/E	N/E
Male sex (No.)	58 (69%)	51 (74%)	0.508	N/E	N/E
Weight (kg)	56.6 (37.0–86.1)	56.7 (37.5–99.2)	0.765	N/E	N/E
Body mass index (kg/m^2^)	21.7 ± 4.1	21.4 ± 4.1	0.653	N/E	N/E
Creatinine clearance (CrCl) at the beginning of therapy^c^ (mL/min)	**53 ± 16**	**58 ± 21**	**0.058**		0.233
Charlson comorbidity index	2 (0–12)	2 (0–12)	0.411	N/E	N/E
Use of inhaled colistin immediately prior to the initiation or after the end of systemic colistin treatment (No.)	**6 (7%)**	**13 (19%)**	**0.029**	**0.331 (0.119–0.925)**	**0.035**
No. of concurrent nephrotoxins used other than intravenous colistin	4 (1–5)	3 (1–5)	0.234	N/E	N/E
Concurrent nephrotoxins (No.)					
Vasopressor	76 (90%)	63 (91%)	0.860	N/E	N/E
Aminoglycoside	11 (13%)	5 (7%)	0.239	N/E	N/E
Polyene	35 (42%)	22 (32%)	0.213	N/E	N/E
Glycopeptide	75 (89%)	64 (93%)	0.459	N/E	N/E
Diuretic	79 (94%)	62 (90%)	0.337	N/E	N/E
Intravenous contrast	13 (15%)	10 (14%)	0.866	N/E	N/E
Use of intravenous colistin loading dose (No.)	46 (55%)	34 (49%)	0.499	N/E	N/E
Maintenance dose of intravenous colistin (mg)	300 (100–1080)	300 (75–900)	0.999	N/E	N/E
Desired target colistin C_ss,avg_ estimated from the maintenance dose^d^ (mg/L)	3.17 (0.98–8.29)	3.18 (0.37–15.56)	0.448	N/E	N/E
Desired target colistin C_ss,avg_^d^ ≥ 2.5 mg/L (No.)	58 (89%)	45 (65%)	0.615	N/E	N/E
Average daily dose of intravenous colistin (mg)	320 (84–1004)	309 (97–900)	0.768	N/E	N/E
Per ideal body weight (mg/kg)	5.7 (1.5–16.0)	5.0 (1.7–15.4)	0.685	N/E	N/E
Per total body weight (mg/kg)	5.8 (1.2–21.0)	5.7 (2.2–18.0)	0.946	N/E	N/E
**Cumulative intravenous colistin dose (mg)**	**4025 (720–33684)**	**5325 (700–94220)**	**0.010**	N/E	N/E
**Duration of intravenous colistin therapy (days)**	**13 (4–134)**	**18 (3**–**156)**	**0.007**	N/E	N/E

Abbreviation: C_ss,avg_, average steady-state plasma concentration; CI, confidence interval; N/E, not estimated.

^a^Mean ± standard deviation or median (range) unless otherwise noted.

^b^Bolded indicate factors significantly associated with nephrotoxicity.

^c^Estimated by the Cockcroft-Gault equation using the actual measured serum creatinine concentration (SCr) if SCr was ≥1 mg/dL and the lower of ideal body weight (IBW) or total body weight (TBW); if the patient’s SCr was <1 mg/dL, SCr was rounded up to 1 mg/dL for CrCl estimation.

^d^Estimated by ${{\rm{C}}}_{{\rm{ss}},{\rm{avg}}}=\frac{{\rm{Daily}}\,{\rm{dose}}\,{\rm{of}}\,{\rm{colistin}}\,{\rm{base}}\,{\rm{activity}}\,({\rm{mg}})}{[(1.50\times {{\rm{CrCl}}}_{{\rm{n}}})+30]}$ where CrCl_n_ is CrCl normalized to body surface area estimated by the Mosteller method at baseline in mL/min/1.73 m^2^.

## Discussion

With the development of the colistin target C_ss,avg_-based dosing algorithm, colistin doses are mostly determined based on the desired target C_ss,avg_ upon the discretion of the clinician treating the patient^15,16^. Considering the narrow therapeutic window of colistin, it is pertinent for clinicians to use the most appropriate desired target C_ss,avg_ for optimal treatment outcomes^13^. Previous studies suggested conflicting evidence regarding the relationship of colistin dose or concentration with treatment outcomes^24–33^. Similar to our current study, several previous studies showed the lack of significant association between treatment outcomes and colistin dose or systemic exposure (Tables 3 and 4)^24–28^. In contrast, other previous studies suggested significantly improved therapeutic effectiveness of colistin at higher doses or systemic exposures^29–33^. Compared to our current study, the patient population included in these previous studies was relatively homogeneous; patients with a specific indication such as burn, bacteremia, and pneumonia were exclusively included in these studies^29–33^. In addition, colistin was administered to patients as a fixed or weight-based dosing (e.g., 2.5–5 mg/kg/day divided into 2–4 times per day) without a desired target C_ss,avg_^29–33^. Similar to treatment outcomes, the relationship between the risk of colistin-associated nephrotoxicity and colistin dose or systemic exposure is controversial^24–33^. Several previous studies reported no significant association between the colistin-associated nephrotoxicity risk and colistin dose or systemic exposure, which is consistent with our current study findings (Table 5)^24,29–31,33^. In contrast, other previous studies suggested significantly increased risk of colistin-associated nephrotoxicity at higher colistin dose or systemic exposures^25,27,32^. While our current study assessed nephrotoxicity using the AKIN criteria, these previous studies evaluated nephrotoxicity based on the RIFLE criteria categorizing nephrotoxicity into risk, injury, failure, loss, and end-stage renal disease^25,27,32^. Overall, these differences in the study population, outcome definition, and colistin treatment strategy between our current study and previous studies might account for the discrepancy in the study finding for the association of colistin dose or systemic exposures with treatment outcomes and the risk of nephrotoxicity.

Although our current study identified no significant predictors for clinical response of colistin therapy (Table 3), several previous studies reported various factors significantly associated with clinical response, including use of colistin loading dose, Charlson comorbidity index, Acute Physiology and Chronic Health Evaluation II (APACHE II) score, presence of severe sepsis, absence of septic shock, sex, absence of nephrotoxicity at the end of colistin therapy, and higher colistin dosages^24,26,29–31,34^. Considering substantial heterogeneity in demographic and clinical characteristics of our study patients with a limited sample size, it might be more challenging in our current study to identify significant predictors for clinical response of colistin therapy. For microbiological outcome, our current study showed significantly higher likelihood of bacterial eradication in patients with bacteremia compared to those with pneumonia and in those receiving IV colistin loading dose (Table 4). Consistently, a previous study using mouse infection models reported colistin to be substantially less effective for the treatment of pneumonia compared to skin and soft tissue infection^12^. This may be because of the possible inadequate antimicrobial exposures in the lungs since pneumonia is often considered a deep tissue infection^12^. The significant association between the use of IV colistin loading dose and microbiological eradication may highlight the importance of adequate initial antimicrobial therapy for optimal treatment outcome. In a previous study by Martinez and colleagues, antimicrobial exposure during the first dose was suggested as the critical factor for the outcome of an infectious disease^35^. This was further supported in a previous *in vitro* study, where the dose-dependent bactericidal activity of colistin was primarily observed during the early phase of therapy^36^. For nephrotoxicity, patients who received inhaled colistin immediately prior to the initiation or after the end of systemic colistin treatment were at a lower risk of developing colistin-associated nephrotoxicity (Table 5), which is consistent with previous study findings^37^. The protective effect of inhaled colistin use may be related to using lower doses of IV colistin; in our study, the median (range) colistin maintenance dose, average daily dose, and the desired target C_ss,avg_ in patients who received inhaled colistin *vs*. in those who did not were 360 mg (220–600 mg) *vs*. 400 mg (120–900 mg), 340 mg (174–525 mg) *vs*. 355 mg (128–773 mg), and 3.37 mg/L (1.59–5.08 mg/L) *vs*. 3.42 mg/L (1.43–9.63 mg/L) (*P* > 0.05; data not shown). Therefore, IV loading dose and inhaled use of colistin may be considered to maximize bacterial eradication and to minimize the risk of nephrotoxicity, respectively.

Our study supports the importance of using IV colistin loading dose in critically ill patients with infections caused by MDR organisms, as suggested by several previous studies^15,18,20,34,38–45^. Although the use of IV colistin loading dose was not significantly associated with clinical response or colistin-induced nephrotoxicity (Tables 3 and 5), bacterial eradication was significantly more likely to occur in patients receiving IV colistin loading dose (Table 4). Similarly, Katip and colleagues suggested higher likelihood of microbiological clearance in patients receiving IV colistin loading dose for the treatment of various infections caused by MDR *Acinetobacter baumannii* compared to those receiving the maintenance dose only (87.9% *vs*. 70.4%, *P* = 0.0006)^39^. As aforementioned, this may highlight the importance of appropriate antimicrobial exposure during the first dose to optimize the treatment outcome of an infectious disease^35^. According to a previous study performed by Kumar and colleagues to evaluate survival after septic shock caused by *Escherichia coli* in a murine model, mortality was significantly increased with hourly delays of adequate antimicrobial therapy^46^. Consistently, Mohamed and colleagues suggested higher likelihood of adequate bacterial killing, defined as ≥3 log killing from the baseline, with colistin loading dose administration, and the extent of bacterial killing was dependent on the amount of colistin loading dose based on the pharmacokinetic-pharmacodynamic modeling and simulation^41,43,45^. The improved bacteriological response with the colistin loading dose may be associated with rapid achievement of the target therapeutic colistin concentrations in plasma^15,20,34,42^. Due to the slow conversion rate of the prodrug (i.e., CMS) to the active drug (i.e., colistin) and the long half-life of colistin (approximately 14.4 h), it can take 2 to 3 days to attain adequate colistin concentrations without a loading dose; administration of a loading dose may reduce the time to reach the target therapeutic colistin concentration within the first few hours^20,34,43^. In contrast to our present study, other previous studies suggested no association between the use of IV colistin loading dose and microbiological response^47,48^. This discrepancy in the association between bacteriological response and the use of IV colistin loading dose may be due to the inclusion of a smaller number of patients in the previous studies compared to our current study and unbalanced sample size between the case (i.e., loading dose used) and the control (i.e., no loading dose) groups^47,48^. The previous population pharmacokinetic study conducted by Grégoire and colleagues in critically ill patients further complicated the issue of IV colistin loading dose^49^. In this study, the estimated half-life of colistin was relatively short (3.1 hours) compared to the half-lives calculated in other previous studies (9.0 to 14.4 hours)^15,20,42,49^, suggesting no need to use IV colistin loading dose^49^. However, caution should be exercised when interpreting the half-life estimates of colistin because of the uncertainty in the fraction converted from CMS to colistin in most of the clinical studies^49^. A recent survey study in physicians, pharmacists, and microbiologists (n = 420) reported only half (52.5%) of the respondents utilized a loading dose always or very often, possibly due to the conflicting evidence available regarding the effect of IV colistin loading dose on treatment outcomes^50^. Large-scale, prospective, clinical studies need to be performed in the near future to determine the effect of IV colistin loading dose on treatment outcomes robustly.

There are some study limitations to be addressed. First, this study may not be adequately powered to identify all of the factors associated with treatment outcomes and nephrotoxicity. Considering the relatively small sample size, caution needs to be exercised when interpreting and applying our study findings to clinical practice. Second, the desired target colistin C_ss,avg_ was retrospectively estimated based on a previously published equation using the actual administered doses^15^. Because plasma colistin concentrations were not measured in our current study, it could not be assessed whether or not our study patients achieved the desired target C_ss,avg_ at the given colistin dose. Third, due to the retrospective nature of this study based on the electronic medical records (EMRs), causality between the tested factors and treatment outcomes or nephrotoxicity risk could not be evaluated. Important information such as APACHE II score and dehydration status was missing for many, if not all, patients in our study. Moreover, clinical microbiology culture and susceptibility data were missing in several patients. Furthermore, colistin susceptibilities in our current study were not determined by the standard broth dilution method proposed by the Clinical and Laboratory Standards Institute (CLSI) and the European Committee on Antimicrobial Susceptibility Testing (EUCAST), which is considered as the current gold standard for antimicrobial susceptibility testing^51,52^. Colistin susceptibility testing was performed in compliance with the standard method at the time of care for patients included in our study (i.e., 2013 to 2014), which was the disk diffusion method to confirm the colistin susceptibility measured by the automated antimicrobial susceptibility testing based on the broth microdilution method (e.g., Vitek 2). Due to the unavailability of the cultured clinical strains currently, colistin susceptibility testing could not be repeated with the standard broth microdilution method proposed by the CLSI and the EUCAST. Although we acknowledge the failure to use the current gold standard antimicrobial susceptibility testing method as a study limitation, our colistin MICs and susceptibility results might be sufficiently reliable because Vitek 2 systems, used to measure colistin MICs in our study patients, are based on the broth microdilution method^53^. Lastly, confounding factors related to human behavior such as prescribing pattern might lead to spurious results for the relationship of tested factors with treatment outcomes or the risk of nephrotoxicity. In the univariate analysis of our current study, larger cumulative doses and longer treatment duration of IV colistin were associated with lower likelihood of clinical cure, bacterial eradication, and nephrotoxicity (Tables 3–5). According to previous studies, these relationships were not considered clinically plausible^24–33^. Our institutional observation revealed this as a unique prescribing pattern. Colistin has been used at the same or higher doses over a prolonged period for patients with inadequate treatment response. In addition, IV colistin was discontinued immediately after the development of clinical signs and/or symptoms of nephrotoxicity even in patients with critical infection. Future clinical studies, preferably prospective studies, are warranted to clarify the effect of colistin doses or systemic exposures on treatment outcomes and safety and to develop and validate an appropriate model to predict colistin exposure in various patient populations for optimal colistin therapy and monitoring.

In conclusion, the desired target C_ss,avg_ of colistin is not associated with treatment outcomes or the risk of colistin-associated nephrotoxicity. However, bacterial eradication is more likely to occur in critically ill patients with bacteremia compared to pneumonia caused by MDR *Acinetobacter baumannii* or *Pseudomonas aeruginosa* and in those receiving a loading dose of IV colistin. Use of inhaled colistin immediately prior to the initiation or after the end of systemic colistin therapy lowers the likelihood of developing colistin-associated nephrotoxicity. Use of an IV colistin loading dose and inhaled colistin near the time of systemic colistin treatment may be considered to maximize therapeutic effectiveness and minimize the risk of colistin-associated nephrotoxicity, respectively.

## Methods

### Study design and patients

This is a retrospective study by reviewing EMRs for patients hospitalized in an ICU at a 2,000-bed tertiary university hospital from January 2013 to December 2014. Adult patients (≥18 years of age) were included if they had a documented acute infection caused by multidrug-resistant bacterial pathogens and received IV colistin for 72 hours or longer. If colistin treatment course was interrupted for more than a week, only the treatment course before interruption was included. Exclusion criteria included pregnancy, breastfeeding, and severe kidney function impairment defined as a baseline serum creatinine concentration (SCr) of ≥4 mg/dL or undergoing renal replacement therapy of any type. Patients who simultaneously received inhaled colistin with IV colistin were excluded from the analysis; however, those who received inhaled colistin prior to the initiation or after the cessation of IV colistin were included. This study was approved by the institutional review board at the study site (Severance Hospital of Yonsei University Medical Center, Seoul, Republic of Korea). All methods were carried out in accordance with relevant guidelines and regulations. The need for informed consent was waived due to the retrospective nature of the study.

### Data collection

The following data were collected from EMRs for patients who satisfied our inclusion and exclusion criteria: demographics including age, sex, weight, and height; disease severity including APACHE II and Charlson comorbidity index; infection-related information including indication, causative organisms identified using the calorimetric Vitek 2 GN ID card (bioMerieux, Durham, NC, USA) in the Vitek 2 automated system, and antibiotic susceptibility of the causative organisms; clinical laboratory test results such as white blood cell count (WBC) and SCr; colistin dosing regimen such as use of a loading dose, the amount of loading dose, maintenance dose and frequency, and duration of therapy; additional antimicrobial agents used besides IV colistin; use of inhaled colistin immediately prior to the initiation or after the cessation of IV colistin; and concurrent use of nephrotoxins other than colistin such as aminoglycosides, glycopeptides, IV contrast, diuretics, polyene, and vasopressors. The antimicrobial MICs of the cultured isolates were determined for clinical practice by the Vitek 2 AST N212 card for nonfermenters in the Vitek 2 automated system (bioMerieux, Durham, NC, USA). The measured MICs were interpreted based on the guidelines published by the CLSI^54,55^. Colistin susceptibility results obtained by the Vitek 2 system were confirmed using the disk diffusion method in accordance with the CLSI standards at the time of patient care^54,55^. The Cockcroft-Gault method was used to estimate CrCl by using the actual measured SCr if SCr was ≥1 mg/dL and the lower of IBW or TBW; if the patient’s SCr was <1 mg/dL, SCr was rounded up to 1 mg/dL^21^.

The desired target colistin C_ss,avg_ was retrospectively estimated using the following equations (Eqs 3 and 4) modified from those suggested in a previous study:

3 $${{\rm{C}}}_{{\rm{ss}},{\rm{avg}}}=\frac{{\rm{Loading}}\,{\rm{dose}}\,{\rm{of}}\,{\rm{colistin}}\,{\rm{base}}\,\text{activity}\,({\rm{mg}})}{2\times {\rm{body}}\,{\rm{weight}}\,({\rm{kg}})}$$

4 $${{\rm{C}}}_{{\rm{ss}},{\rm{avg}}}=\frac{{\rm{Daily}}\,{\rm{dose}}\,{\rm{of}}\,{\rm{colistin}}\,{\rm{base}}\,{\rm{activity}}\,({\rm{mg}})}{[(1.50\times {{\rm{CrCl}}}_{{\rm{n}}})+30]}$$

where body weight is the lower of IBW or TBW in kg, and CrCl_n_ is CrCl normalized to body surface area at baseline in mL/min/1.73 m^2^ ^15^. Body surface area was estimated by the Mosteller method^22^.

Treatment effectiveness was evaluated based on clinical cure and microbiological eradication. Clinical cure was defined as the resolution of infection signified by clinical improvement such as temperature <37 °C for ≥72 hours, WBC < 12,000 cells/mm^3^, no radiologic evidence of active infection, no other clinical infectious signs and symptoms, and the lack of re-occurrence of the same infection during the hospitalization period. Microbiological eradication/cure was defined as the documented elimination of the original causative bacterial pathogen from the site of isolation through follow-up culture studies during or at the end of colistin therapy. Otherwise, the cases were classified as either clinical or microbiological failure. Nephrotoxicity was defined as an increase in SCr by 0.3 mg/dL or 1.5- to 2-fold increase in SCr from baseline according to the Acute Kidney Injury Network (AKIN) criteria^23^. Nephrotoxicity that occurred at ≥48 hours after the initiation of IV colistin therapy was deemed to be associated with colistin.

### Statistical analysis

All statistical analyses were performed using SPSS Statistics 23.0 (IBM SPSS Statistics for Windows, Version 23.0, Armonk, NY: IBM Corp.). Categorical variables were analyzed using the chi-square or Fisher’s exact test. Continuous variables were compared between the treatment success and failure groups using the two independent sample Student’s t test for normally distributed data or the Mann-Whitney U test for non-normally distributed data based on the Kolmogorov-Smirnov normality test results. Factors evaluated for the association with clinical response in the univariate analysis included all of the demographic and clinical characteristics of study patients. For microbiological eradication, bacterial susceptibility in addition to all of the factors tested for the association with clinical response were evaluated. For nephrotoxicity, the followings were tested in addition to the factors assessed for clinical response: the number of concurrently used nephrotoxins; and use of a concurrent vasopressor, nephrotoxic antimicrobial agents, diuretics, and an IV contrast media.

Univariate analyses were performed to compare patients with and without clinical cure, microbiological eradication, and nephrotoxicity. Based on the univariate analysis results, multiple logistic regression was performed using a stepwise forward method by evaluating the factors identified as significantly associated with clinical cure, microbiological eradication, and nephrotoxicity, respectively. In order to ensure the inclusion of all potentially pertinent factors, variables with *P* < 0.10 from the univariate analysis and clinical plausibility were assessed in the multiple logistic regression. Statistical significance in the multivariable logistic regression was defined as *P* < 0.05.

## Data Availability

The datasets generated and/or analysed during the current study are not publicly available due to the inclusion of private medical information at our institution, but may be available from the corresponding author on reasonable request.
